# Targeted alignment and end repair elimination increase alignment and methylation measure accuracy for reduced representation bisulfite sequencing data

**DOI:** 10.1186/s12864-016-2494-8

**Published:** 2016-02-27

**Authors:** Saurabh Baheti, Rahul Kanwar, Meike Goelzenleuchter, Jean-Pierre A. Kocher, Andreas S. Beutler, Zhifu Sun

**Affiliations:** Division of Biomedical Statistics and Informatics, Mayo Clinic, Rochester, MN 55905 USA; Department of Medical Oncology, Mayo Clinic, Rochester, MN 55905 USA; Charité - Universitaetsmedizin Berlin, Berlin, Germany

**Keywords:** TRACE-RRBS, Reduced representation bisulfite sequencing, RRBS, DNA methylation, RRBS alignment, Methylation measure accuracy

## Abstract

**Background:**

DNA methylation is an important epigenetic modification involved in many biological processes. Reduced representation bisulfite sequencing (RRBS) is a cost-effective method for studying DNA methylation at single base resolution. Although several tools are available for RRBS data processing and analysis, it is not clear which strategy performs the best and there has not been much attention to the contamination issue from artificial cytosines incorporated during the end repair step of library preparation. To address these issues, we describe a new method, Targeted Alignment and Artificial Cytosine Elimination for RRBS (TRACE-RRBS), which aligns bisulfite sequence reads to *MSP1* digitally digested reference and specifically removes the end repair cytosines. We compared this approach on a simulated and a real dataset with 7 other RRBS analysis tools and Illumina 450 K microarray platform.

**Results:**

TRACE-RRBS aligns sequence reads to a small fraction of the genome where RRBS protocol targets on and was demonstrated as the fastest, most sensitive and specific tool for the simulated dataset. For the real dataset, TRACE-RRBS took about the same time as RRBSMAP, a third to a sixth of time needed for BISMARK and NOVOALIGN. TRACE-RRBS aligned more reads uniquely than other tools and achieved the highest correlation with 450 k microarray data. The end repair artificial cytosine removal increased correlation between nearby CpGs and accuracy of methylation quantification.

**Conclusions:**

TRACE-RRBS is fast and more accurate tool for RRBS data analysis. It is freely available for academic use at http://bioinformaticstools.mayo.edu/.

**Electronic supplementary material:**

The online version of this article (doi:10.1186/s12864-016-2494-8) contains supplementary material, which is available to authorized users.

## Background

DNA methylation, the addition of a methyl group to the 5’ position of the cytosine, is one of the major epigenetic control mechanisms for gene regulation, cellular differentiation, embryogenesis, X chromosome inactivation, genomic imprinting, and tumor genesis [1]. The methylated cytosine (C) is resistant to bisulfite treatment while the treatment converts unmethylated cytosine residues to uracil, which subsequently changes to thymine (T) in PCR reaction. This property (the artificial C/T transition) is used to interrogate the cytosine methylation status at base level for quite some time but it was not until the advent of next generation sequencing technology that methylome-wide studies have become possible in recent years [2]. Bisulfite methylation sequencing (BS-Seq) can be conducted for all CpGs in the genome (whole genome bisulfite sequencing, WGBS) or for selected regions of interest to reduce cost and complexity such as reduced representation bisulfite sequencing (RRBS) and Agilent SureSelect Methyl-Seq Kit (www.agilent.com). RRBS is the most commonly used approach, which applies *MSP1*, a restriction endonuclease, to cut DNA at CCGG motif sites into fragments and then sequence the regions enriched with CpG sites in the genome. Although the approach only targets ~2 million or 7-10 % of CpGs in the genome, these CpGs are highly enriched in the most important regulatory regions of the genome such as CpG islands, CpG island shore, promoters, and gene body, which makes it one of very cost-effective approaches to analyze DNA methylation [2–5].

A number of tools have been developed to analyze RRBS or bisulfite sequencing data. BSMAP [6] is a bisulfite sequencing alignment tool that aligns a bisulfite read to the reference genome directly without reference genome conversion through enumerating all C-to-T combinations within a user-defined seed length of the read. BISMARK [7] uses Bowtie [8] or Bowtie 2 [9] as an aligner. Both reference genome and sequence reads are converted mimicking bisulfite-treated DNA, i.e., changing C to T for forward and G to A for reverse strands. Sequence reads that produce a unique best alignment from the four alignment processes against the bisulfite genome are then compared to the normal genomic sequence and the methylation state of all cytosine positions in the read is determined. Similarly, BS-SEEKER2 [10] aligns the converted reads to the four pre-built indexes built separately from a three-letter converted genome. It uses Bowtie 2 [9] or SOAP as the aligner of choice. LAST [11, 12] is an enhanced aligner that uses seed extension approach similar to be the one used by NCBI BLAST [13]. It has three steps to get the reads aligned to the reference genome, which includes indexing the genome, finding alignments, and then resolving multiple mapping reads through estimating the probability that each is the correct one so that only alignments with low mismatch probability are retained. MethylCoder [14] uses GSNAP [15] or Bowtie 2 [9] to do a primary mapping of in-silico converted reads (that is, all Cs in the reads are converted to Ts) onto a converted reference genome. The reads that fail to map onto the converted genome will be remapped again in their original forms onto the original reference genome. Novoalign (http://www.novocraft.com) basically uses the statistical model from SOAP, which is designed for heterogeneous samples and uses a traditional 4 base model to call the consensus sequence and then for each cytosine it reports the % of bases that are methylated (i.e. not converted to thyamine). BRAT-BW [16] uses the same strategy as Bismark and BS-seeker2. To align the data, two FM-indexes are built from the original reference genome: one converted from all Cs to Ts for the forward strand and another from Gs to As for the reverse strand. Original reads with Cs converted to Ts are mapped to the first index, and the reverse-complements of the reads with Gs changed to As are mapped to the second index. It also uses a multi-seed approach by attempting to align a read starting from different locations within the read. The further details and feature comparisons of these tools are summarized in Additional file 1: Table S1.

In spite of these multiple tools, their relative performance of analyzing RRBS data is not well established. Additionally, several issues unique to RRBS have not been addressed adequately: (1) RRBS reads are generated from known regions in the genome (with CCGG motif) and aligning RRBS reads to whole genome is less efficient and more likely to have misalignment as the reduced complexity of both reference and reads from 4 to 3 letters. (2) Significant amount of RRBS reads (10–15 %) may be contaminated by sequence adapters as the selected fragments range from 40 to 250 bases to accommodate the shorter fragments with rich CpGs (i.e., fragments shorter than sequence reads would be sequenced into adapter sequences). This makes adapter trimming an indispensable step before alignment. All above tools need this pre-processing well conducted for good alignment [17]. However, when adapter sequence within a read is short (such as a few bases), adapter trimming gets difficult and true biological bases can be trimmed off inadvertently. (3) *MSP1* cuts DNA between two Cs at CCGG motif. This is followed by end repair, A-tailing, and adapter ligation before bisulfite conversion. The end repair incorporates artificial CG at the end of a read. When a RRBS fragment is shorter than a sequence read, the incorporated Cs (generally not methylated) can remain and become part of DNA fragment sequence (adapter trimming does not remove the end repair bases), which can bias a methylation estimate at these positions.

To deal with the aforementioned issues, we have developed a unique RRBS analytical tool called “TRACE-RRBS” (Targeted Alignment and Artificial Cytosine Elimination for RRBS). Unlike other methods, TRACE-RRBS first creates a reference genome by digitally digesting reference sequence by *MSP1* into DNA fragments and only the fragments that are within RRBS size selection range are kept, mimicking a true RRBS experiment. The targeted alignment increases alignment speed and accuracy. Adapter sequences are attached to the ends of the digital digested fragments for later removal so no adapter trimming is needed during alignment. Furthermore, artificial end repair C bases are recorded for exclusion during methylation data extraction.

## Results

### TRACE-RRBS algorithms

#### Aligning reads to their origin of the genome

**Fig. 1:**
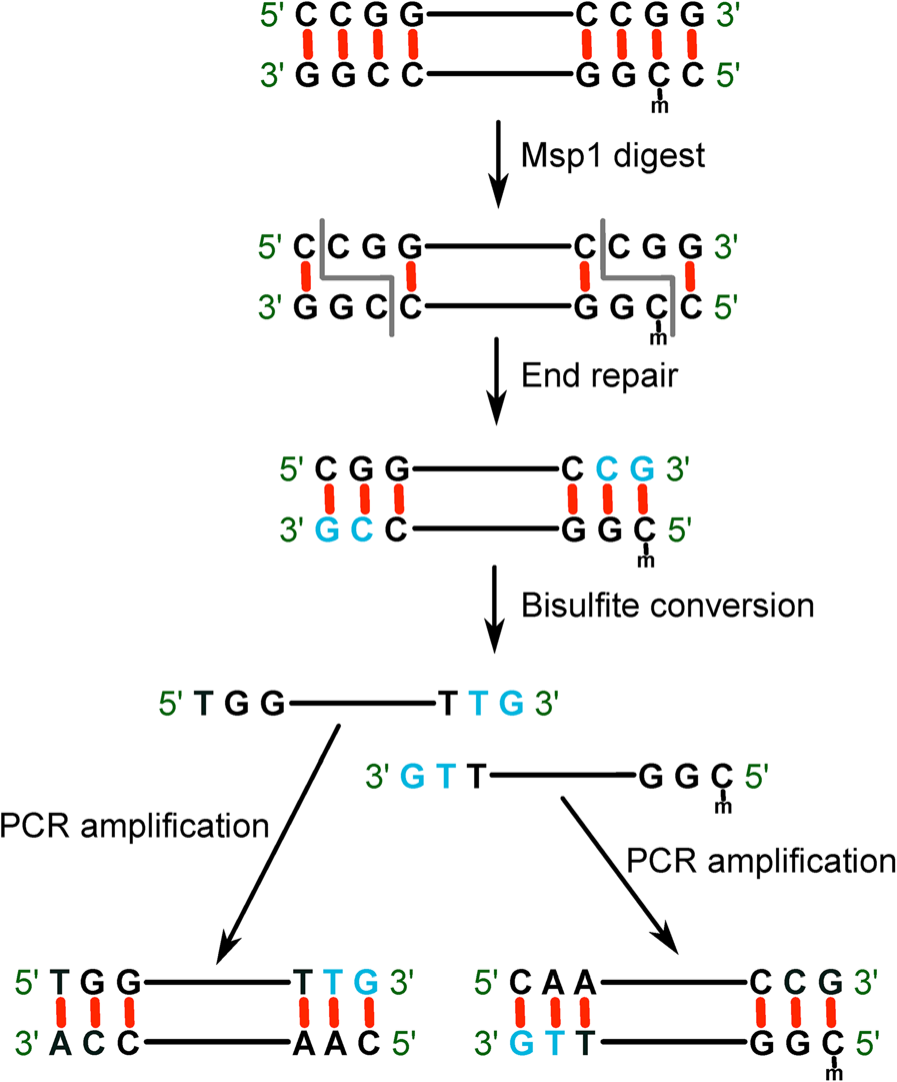
RRBS concept and workflow. Genomic DNA is first digested by MSP1 which cuts at CCGG sites. These fragments are end-repaired, A-tailed, and adapter-attached and then bisulfite treated. The unmethylated C is converted to U (T) and methylated C is unchanged. The blue bases are from media and are not real biological signal that needed to be removed

The unique characteristic of RRBS is to cut the genome between two Cs of the motif CCGG using the *MSP1* enzyme. The fragmented genome is then bisulfite treated and sequenced. Each of the sequenced fragments begins with CGG and the resulting sequenced reads contain at least one CpG, i.e. the leading two base pairs of the read (Fig. 1). In the alignment step, all the cytosines on the reference genome are converted into thymines (forward strand). This in-silico bisulfite conversion of the genome results in a three-letter genome and the reverse complementarity of the two strands is lost. The cytosines on the reads are also converted into thymines before aligning against the in-silico converted genome. Since we already know where RRBS fragments come from the genome, we can use this information to improve the accuracy of the alignment step for two simple reasons: First, C/T conversion in the reference genome and reads reduce sequence complexity and the chance of aligning to multiple or incorrect locations increases if we align reads to whole genome. However, if we create a reference genome only from the regions the RRBS reads arise from, this chance would be significantly reduced and alignment accuracy would be increased. Secondly, *MSP1* fragments appropriate for RRBS only account for about 1 % human genome. Aligning reads to this small subset of the genome significantly reduces alignment space and increases alignment speed.

#### Dealing with adapter sequence and end-repair incorporated cytosine

A critical preprocessing step is the adapter trimming for RRBS as a significant number of fragments can be shorter than sequence reads. To cut adapter sequence, we need to specify a minimum number of bases in a read that matches to an adapter sequence. Setting this number high would leave partial adapter sequence intact and reduce alignment rate and possibly introduce non-biological bases. Specifying a too small number (such as 1–3) is less specific and may remove biological signals. Additionally, the artificial trailing Cs are incorporated into sequence reads during the end repair and A tailing step (Fig. 1, blue bases) and these need to be removed during alignment or methylation level estimation step to avoid biased measurement. Trim Galore (http://www.bioinformatics.babraham.ac.uk/projects/trim_galore/) and BSeQC [18] are the tools that can conduct end repair base removal. The former is a wrapper script for Cutadapt [19] and FastQC (http://www.bioinformatics.babraham.ac.uk/projects/fastqc/) before alignment, which is a separate process and adapter trimming can be difficult for short sequence as discussed above. Removal of additional bases for pair-end sequencing can be tricky as it can affect subsequent RRBS read alignment. For example, removing two additional bases from the beginning of the read 2 (complementary reads to the original forward and reverse strands) would remove CGG tag that is used to search for indexed CCGG motif in the reference genome causing the reads to remain unaligned in RRBSMAP. BSeQC is a quality control tool only for aligned bam file of bisulfite sequencing for potential bias removal including the end repair base. TRACE-RRBS deals with all the issues in a single pipeline where no adapter trimming and explicit end repair C removal are needed as detailed below.

### TRACE-RRBS implementation

**Fig. 2:**
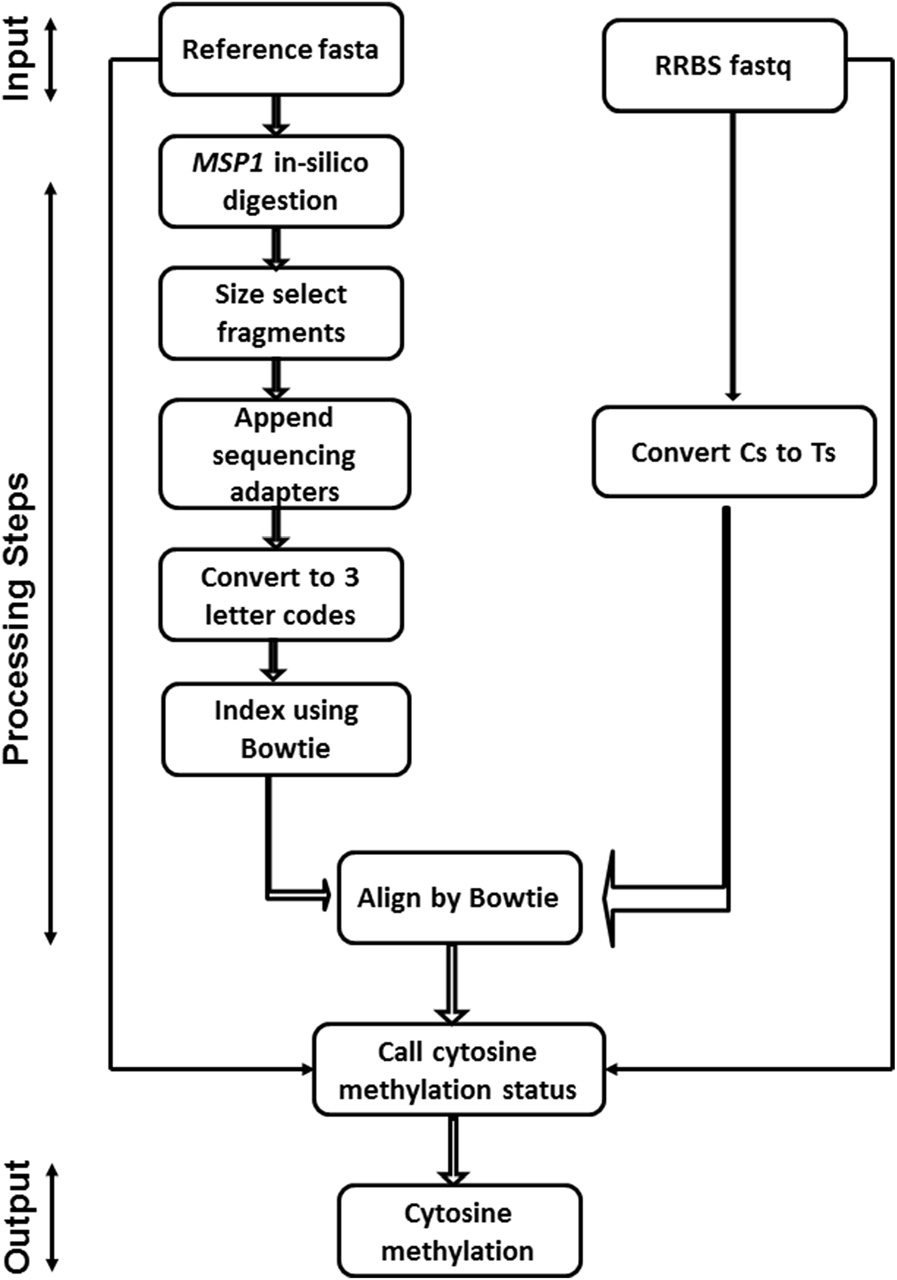
TRACE-RRBS Flowchart. The reference genome in fasta is first digitally digested into fragments mimicking a real RRBS experiment. These fragments are size selected, end repaired, A-tailed and an adapter annealed. They are further indexed using Bowtie 2. Both sequence reads and the reference fragments are converted fully from Cs to Ts for forward and Gs to As for reverse strand before alignment by Bowtie 2. After alignment TRACE-RRBS parses the bam, removes an adapter and incorporated Cs, and compares with unconverted sequence for methylation calculation

TRACE-RRBS (Fig. 2) has three different major components. In the first part it takes an input reference fasta file and conducts an in-silico digestion for the *MSP1* motif [CCGG]. All the genomic positions containing a CCGG motif are clipped which results in a collection of variable sized DNA fragments. An artificial CG is added to the ends of each fragment. This corresponds to the end repair step of the library preparation protocol. The fragments are then size selected based on user input as per RRBS protocol (for example 40-250 bps), followed by the attachment of sequencing adapters. They are then in-silico bisulfite converted by replacing Cs to Ts for the forward and Gs to As for the reverse strand. These “post-bisufite” fragments are indexed using Bowtie 2 [9] and used for the alignment step. The positions of added artificial CG and adapter sequences are tracked for later removal as they are not biological sequences.

The second part is the alignment of RRBS reads against the reference genome created from the previous step using Bowtie 2 [8, 9]. For this step we convert the RRBS reads into a 3-letter code by converting Cs to Ts for forward reference alignment and Gs to As for reverse reference alignment. Similar conversion is performed for the second read if it is pair-end sequencing. By converting an RRBS read to a 3-letter code we lose its methylation information, but gain the ability to align it against the 3-letter “post-bisulfite” converted fragment. Once the read is aligned to the “post-bisulfite” fragment we know its exact origin, we can then traverse the overlap of the read and the fragment, looking for positions that correspond to a C on the corresponding “pre-bisulfite” fragment. For such a position we can look into the corresponding pre-converted read to see whether the base is a C (methylated cytosine) or a T (unmethylaed cytosine). This strategy is used to determine the methylation state of a cytosine in the next step.

The final part is the methylation-calling step for genomic cytosines using the aligned RRBS reads. All the reads that align to multiple fragments are discarded. For the case of a read aligning to a single fragment, maybe multiple times, we look if there is an alignment where the beginning of the read is aligned with the beginning of the fragment. We use this particular alignment of the read in the subsequent steps. For each cytosine of a fragment we construct a pileup of the aligned pre-converted reads and report the fraction of Cs observed in it as the methylation ratio. Noted is that the adapter and incorporated sequences are ignored in this step.

TRACE-RRBS is implemented using platform independent Java language for digital reference genome digestion, RRBS fragment reference genome preparation with annealed adapter, post-alignment processing and methylation estimation. The alignment tool incorporated is Bowtie 2 [9]. Some libraries from Picard (http://broadinstitute.github.io/picard/) are used and incorporated in the TRACE-RRBS package.

### Performance comparison for the simulated RRBS data

Tables 1 and 2 summarize the alignment and methylation call statistics for the 8 tools, respectively. TRACE-RRBS demonstrated as the fastest aligner among all using less than 0.5 h, with BSMAP (RRBS mode) the second. TRACE-RRBS also aligned the highest percentage of reads (88.59 %, sensitivity) with the highest accuracy (87.45 %, specificity).

**Table 1:** Alignment performance comparison of simulated RRBS reads (10 Million)

Tool	Alignment^a^ time (hours)	Memory usage (GB)	%Unique reads	%Correct mapped
BISMARK	0.53	8.4	81.03 %	80.79 %
BRAT-BW	2.89	12.69	80.46 %	74.11 %
BSMAP	0.34	2.78	87.20 %	85.92 %
BS-SEEKER2	0.66	5.3	73.28 %	71.85 %
LAST	0.71	16.1	78.11 %	77.96 %
TRACE-RRBS	0.26	8.76	88.59 %	87.45 %
METHYLCODER	2.5	25.83	62.38 %	44.82 %
NOVOALIGN	1.19	16.1	86.59 %	86.59 %

^a^Alignment time doesn’t include the genome preparation time

**Table 2:** Methylation extraction and calculation of simulated RRBS reads

Tool	Time (hours)	Memory usage (GB)	%Recall^a^	R^2^ with Truth from Simulator
BISMARK	1.08	5.78	81.03 %	0.844
BRAT-BW	2.89	2.5	80.46 %	NA
BSMAP	0.4^b^	3.14^b^	87.20 %	0.864
BS-SEEKER2	0.54	6.1	73.28 %	0.869
LAST	--	--	78.11 %	0.757
TRACE-RRBS	0.17	7.68	88.59 %	0.988
METHYLCODER	--	--	62.38 %	0.645
NOVOALIGN	1.19	6.2	86.59 %	0.878

^a^Number of CpGs with at least 1X coverage divided by total expected CpGs

^b^time and memory for chromosome 1 as BSMAP script takes a huge amount of memory for whole genome

*NA* no available script to extract methylation data for the comparison; -- the tools have their methylation extraction script but was not able to be used in this case and an in-house script was used

We further compared the estimated methylation ratio with the truth for each of the tools using the square of the Pearson correlation (R-squared) for measurement accuracy. TRACE-RRBS achieved the highest R^2^ 0.99 while all other tools were below 0.90. The recall rate (the number of CpGs with at least 1X over the total expected CpGs) of TRACE-RRBS was also the highest (88.6 %) and methylcoder was the lowest (62.4 %).

### Performance comparison for MCF7 cell line and Illumina 450 data

For this dataset, we compared the execution time, memory usage, mappability and correlation with Illumina Infinium Human45k microarray. The run time (3.2 h) and uniquely aligned reads (49.4 %) of TRACE-RRBS were comparable with BSMAP RRBS module and BS-SEEKER 2. NOVALIGN and BISMARK also had the similar aligned reads; however, the alignment times were 3 to 6 times more (10.9 and 24.5 h, Table 3). At 10X coverage, TRACE-RRBS captured 2.4 million CpGs, the third highest after METHYLCODER and LAST but significantly higher than BISMARK (1.87 M), BS-SEEKER2 (1.98 M), BSMAP (2.11 M), and NOVOALIGN (2.14 M). In correlation comparison with 450 K microarray data, TRACE-RRBS data has the highest correlation along with BS-Seeker2 (0.95, Table 3, Additional file 1: Figure S1). Identical correlation was obtained for BISMARK, BSMAP and NOVOALIGN (0.91). However, the correlation for Methylcoder was the lowest at 0.64 even though it had the highest number of CpGs at 10X coverage. This low correlation was partly contributed by the methylation extraction script within the package as when we used our extraction method this correlation was significantly increased (nearly 0.8) although it was still lower than others. This may also explain the low correlation for the simulated data.

**Table 3:** Performance comparison of MCF7 RRBS reads

	TRACE-RRBS	BISMARK	BRAT-BW	BS-SEEKER2	BSMAP	METHYL-CODER	NOVO-ALIGN	LAST
Run Time (hours)^a^	3.2	10.89	9.1	3.6	3.19	14.4	24.5	11.2
Memory Usage (GB)	8.4	12.69	2.78	5.3	6.1	18.76	13	16.1
%Unique Reads	49.4	48.1	40.7	48.5	49.6	41.2	49.5	45.5
#C@10X (million)	2.38	1.87	NA	1.98	2.11	2.72	2.14	2.41
Correlation with 450 K chip (R^2^)	0.95	0.91	NA	0.95	0.91	0.64	0.91	0.84

^a^Alignment time only

*NA* no associated script to extract methylation data

### TRRACE-RRBS corrects the end repair C bias

**Fig. 3:**
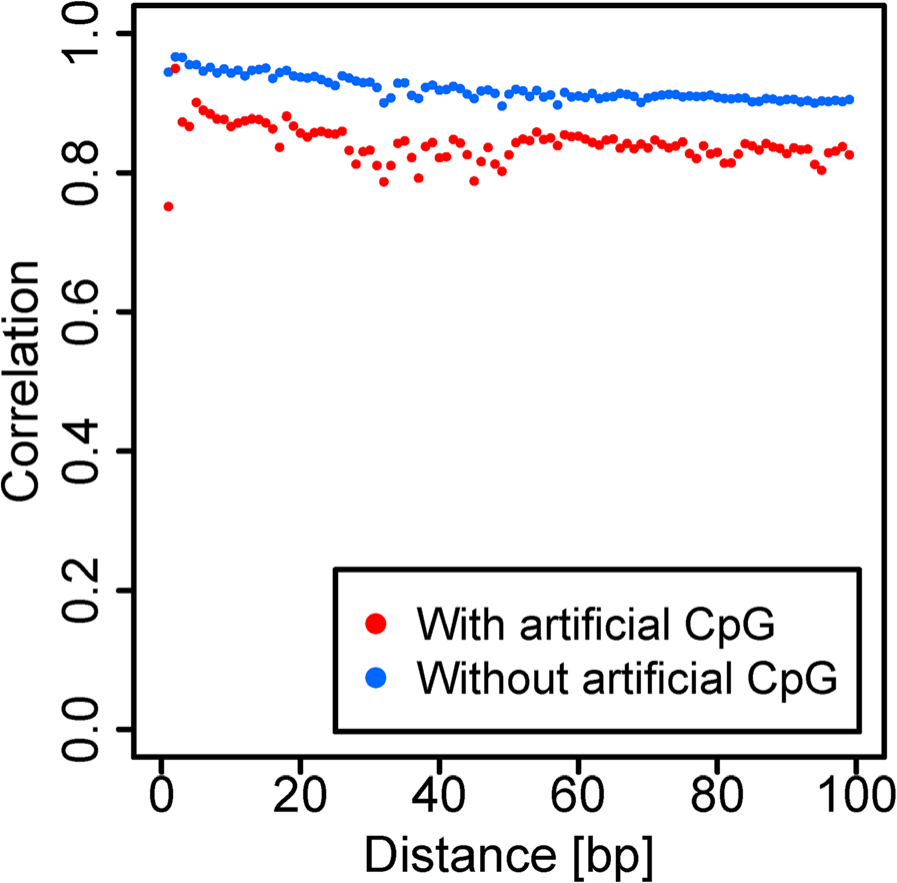
Methylation ratio auto-correlation of CpGs at fixed distances before and after end repair C removal. Autocorrelation plot shows the correlation among neighbouring CpG sites (y-axis) dependents on the distance between two sites compared (x-axis in bp). The red dots represent the case where the artificially added CpGs are included in the cytosine methylation calculation. The blue dots represent the case when they are excluded. Significantly improved auto-correlation is observed after artificial Cs are removed

From the simulated and the real data, TRACE-RRBS achieved the highest correlation with the simulated true methylation and the Illumina 450 k platform, partly as a result of end C removal as it is an integral part of analysis by TRACE-RRBS. To further demonstrate the improvement, we conducted the methylation auto-correlation analysis as performed previously [20] for CpGs at distance from 1 to 100 bps before and after the end repair C removal. The closer the nearly CpGs are, the higher correlation are expected as CpG methylation in the genome is in cluster fashion. As seen in Fig. 3, the methylation level without the end repair C removal had higher variability and lower correlation (the red dots); however, this was significantly improved after the end repair C removal (the blue dots).

## Discussion

RRBS is the most commonly used bisulfite sequencing application in biological and medical research because of its affordability and significant enrichment in the genic and regulatory regions in the genome. Many tools have been developed; however, the performance data are often inconsistent. As RRBS reads come from known genomic regions, it is less effective and error prone to align them to whole genome. We hypothesize that aligning RRBS reads to the *MSP1* cut fragments and conducting end repair C removal improve alignment speed, accuracy, and methylation measurement. As shown from our evaluation and comparison, these have been demonstrated the case. TRACE-RRBS performs on the top among most measures. It does not need an adapter trimming step yet conduct end repair C removal to avoid data bias. On the other hand, as the tool creates RRBS reference with sequence adapter attached, users may need to recreate the reference again if different adapter sequences are used, although not common. This is fairly easy with the enclosed setup script.

TRACE-RRBS is not library preparation dependent. To confirm this, we further downloaded a RRBS and Infinium 450 K microarray data for MCF7 from the Encode project (http://hgdownload.cse.ucsc.edu/goldenPath/hg19/encodeDCC/) and conducted the similar analyses as the presented real data. Unsurprisingly we obtained very similar results (Additional file 1: Table S2). Again, TRACE-RRBS took the least amount of time for alignment along with BSMAP, had intermediate memory usage, and obtained the highest methylation correlation with the methylation microarray data.

Adapter trimming is almost mandatory for RRBS data processing as per routine RRBS protocol DNA fragments range from 40 to 220 bps and sequence reads are generally greater than 50 bases. The longer reads than sequenced fragments means a certain percentage of sequence reads are contaminated with adapter sequences that need to be trimmed off for better alignment. There are several ways to deal with adapters. (1) Dynamic trimming of sequence reads according to adapter sequences to remove adapters. (2) Hard trimming to remove a fixed length of sequence from read ends. Both are evaluated previously by Chatterjee et al. [17] and they validated that adapter trimming is a critical step. However, they also found that if adapter trimming is not done correctly, it can affect alignment and methylation ratio estimation. For example, older version of BSMAP (1.02) has –c option in alignment that led unexpected 5’ single-base truncation and a 10 bp 3’ truncation, which affected alignment dramatically. Although the option is removed in the later versions of the tool, it illustrates the detrimental effect of improper adapter trimming. Hard trimming too aggressively may remove useful sequences and result in a slight change of methylation estimate. TRACE-RRBS takes a very different approach to handle adapter sequences. Instead of trimming adapter sequences from sequence reads, adapter sequences are attached to each *MSP1* digested reference sequence fragment at both ends allowing reads with adapters to correctly align. As the exact attaching locations of adapter sequences are known, TRACE-RRBS can ignore them precisely (not physical but soft trimming), which can contribute to its better methylation estimate.

As introduced in background section, most RRBS analysis tools create two converted reference genomes for converted read alignment. The conversion reduces sequence complexity from 4 to 3 letters and the chance of multiple mapping due to less uniqueness of reference genome increases. Aligning RRBS reads to converted whole genome with high repetitive regions for species like human has a higher chance of multiple mapping than aligning to limited MSP1 digested regions. We assessed the uniqueness of the DNA fragments in the range of 40 to 250 bps generated from MSP1 digestion (hg19) and found 2.27 % have exact sequences even before converting all Cs to Ts but after the conversion, the increase is very minimal to 2.59 %. When sequence reads come from these regions, they would have additional chance to align to other genomic regions (the remaining 99 % not from MSP1 digestion) as multi-mapping.

## Conclusion

In summary, we have developed a new method and software package, TRACE-RRBS, for fast and accurate mapping of RRBS reads and determination of the CpG methylation status of each cytosine locus. It is easy to use and the output of our tools is the standard BAM format for alignment. The methylation calls are in an easy-to-interpret format. Our comparisons with respect to other popular bisulfite sequencing tools have showed that TRACE-RRBS outperforms in most measures on the real and simulated data.

## Methods

### Other tools compared and computation environment

We tested and compared our algorithm and tool with BSMAP (v 2.74), BS-SEEKER2 (v2.0.5), BISMARK (0.10.1), BRAT-bw (v2.0.1) Methylcoder and LAST (v389). The features of these tools are summarized in Additional file 1: Table S1.

The mapping and methylation quantification runs were performed on a Linux server with 32 cores (Intel(R) Xeon(R) CPU, X5650, 2.67GHz) and with 48 Gb RAM running 64bit Red Hat 4.4.7-4. We ran all the tools using their default settings and CPU threads (thread = 1). BS-SEEKER2, BSMAP, NOVOALIGN and TRACE-RRBS have built-in functions to remove adapters, while BISMARK, BRAT-BW, METHYLCODER and LAST do not. As a result we performed an additional step for adapter trimming (by running cutAdapt) for all these tools.

### Test datasets

A simulated RRBS data with 10 million reads was generated using our tool sim-rrbs simulator. The reads were 50 bp long and randomly simulated from the human reference genome (hg19) downloaded from UCSC with methylation probability of 20 % at each CpG site. The reads were selected from the pool of 30 to 250 bps fragments with a mean fragment length of 70. Once the reads were generated, we replaced the quality scores with a real RRBS run for a human sample.

For a real RRBS dataset, we downloaded MCF7 breast cancer cell line with about 50 million single-end reads from Illumina Genome Analyzer II (Gene Expression Omnibus (GEO) accession number [GSE27003]). All the tools were run on their default settings. Only the uniquely mapped reads were used for performance evaluation.

For each tool, following performance measures were compared: (1) Alignment time and computational resources. (2) Alignment efficiency and accuracy. These were measured by percentage of reads aligned back to the genome and their uniqueness and correctness. Only reads mapped to the exact position from their origin were counted. (3) Estimated methylation ratio. Only CpG positions were used for this measure. Methylation ratio was calculated by dividing the number of methylated C by total number of C at the C position of a CpG. For the simulated data we compared this ratio against the truth, i.e., the methylation level we set in the simulation. For the real dataset of MCF7, we compared with the data from Illumina 450 K array platform downloaded from GEO with accession number GSE44837.

All data used in this study were either simulated or downloaded from public database GEO for cell lines with no human subjects or animals involved and therefore no consent and IRB approval were necessary.

## Availability of supporting data

The simulated data is available at our website (http://bioinformaticstools.mayo.edu/). All other data used in this study are readily available from GEO with accession numbers indicated in the method section.

## Additional files

Additional file 1: Table S1. Summary of different aligners/tools for RRBS (WGBS). **Table S2.** Tool performance comparison of MCF7 RRBS and 450 K array generated by ENCODE project. **Figure S1.** Correlation plot between 450 K array and RRBS from comparison tools for MCF7. Pair-wide scatter plot and correlation between Illumina 450 k array (y-axis) and RRBS analysed by each tool (X-axis) for MCF7 cell line. The highest correlation is seen in TRACE-RRBS (TRACE). (DOCX 477 kb)

## Abbreviations

BS-Seq: bisulfite methylation sequencing
GEO: gene expression omnibus
RRBS: reduced representation bisulfite sequencing
WGBS: whole genome bisulfite sequencing
