# Supplementary material for: Targeted alignment and end repair elimination increase alignment and methylation measure accuracy for reduced representation bisulfite sequencing data
Source: BMC Genomics. 2016 Feb 27;17:149. doi: 10.1186/s12864-016-2494-8 (PMC4769831; doi:10.1186/s12864-016-2494-8)
Supplement: Additional file 1: Table S1. — Summary of different aligners/tools for RRBS (WGBS). Table S2. Tool performance comparison of MCF7 RRBS and 450 K array generated by ENCODE project. Figure S1. Correlation plot between 450 K array and RRBS from comparison tools for MCF7. Pair-wide scatter plot and correlation between Illumina 450 k array (y-axis) and RRBS analysed by each tool (X-axis) for MCF7 cell line. The highest correlation is seen in TRACE-RRBS (TRACE). (DOCX 477 kb) [file 12864_2016_2494_MOESM1_ESM.docx]

Supplementary information

ADDITIONAL Table 1: Summary of different aligners for RRBS (WGBS)

|  | **Trace-RRBS** | **Bismark** | **Brat-bw** | **BS-Seeker2** | **Bsmap** | **Methylcoder** | **Novoalign** | **last** |
| --- | --- | --- | --- | --- | --- | --- | --- | --- |
| **Version** | 0.1.0 | 0.10.1 | 2.0.1 | 2.05 | 2.74 | 0.14.1 | 2.08.01 | 389 |
| **Language** | java | perl | C++ | python | C++ | python | C++ | C++ |
| **Aligner** | Bowtie | Bowtie | Reference hashing and wildcard matching | Bowtie | SOAP | GSNAP/Bowtie | Novoalign | LAST |
| **Sequencing Technology** | Base space | Base space | Base space | Base space | Base space | Base space | Base space | Base space |
| **Reference/Read conversion** | Both reference and reads | both reference and reads | both reference and reads | both reference and reads | No | Reference | Both reference and reads | Both reference and reads |
| **Alignment strategy** | Msp1 digitally digested DNA fragments or whole genome | Whole genome | Whole genome | MSPI digitally digested DNA fragments or whole genome | Index of Msp1 cut motif sites or whole genome | Whole genome | Whole genome | Whole genome |
| **Sequencing Mode** | Single-end and paired-end | Single-end and paired-end | Single-end and paired-end | Single-end and paired-end | Single-end and paired-end | Single-end and paired-end | Single-end and paired-end | Single-end and paired-end |
| **Output** | Mapping output including tools for CpG quantification | Mapping output including tools for CpG quantification | Mapping Output txt-> Non-standard SAM | Mapping output including tools for CpG quantification | Mapping output including tools for CpG quantification | Mapping output | Mapping output including tools for CpG quantification | Mapping Output txt-> Non-standard SAM |
| **WGBS** | Yes | Yes | Yes | Yes | Yes | Yes | Yes | Yes |
| **RRBS** | Yes | Yes | Yes | Yes | Yes | Yes | Yes | Yes |
| **End repair removal for RRBS** | Yes | No | No | No | No | No | No | No |

Additional Table 2: Tool performance comparison of MCF7 RRBS and 450K array generated by ENCODE project

|  | **TRACE-RRBS** | **BISMARK** | **BRAT-BW** | **BS-SEEKER2** | **BSMAP** | **METHYL-CODER** | **NOVO-ALIGN** | **LAST** |
| --- | --- | --- | --- | --- | --- | --- | --- | --- |
| Run Time (hours)* | 2.2 | 7.6 | 6.4 | 2.5 | 2.2 | 10.1 | 17.2 | 7.8 |
| Memory Usage (GB) | 5.9 | 8.9 | 1.9 | 3.7 | 4.3 | 13.1 | 9.1 | 11.3 |
| %Unique Reads | 46.5 | 44 | 41.8 | 44 | 46.8 | 45.7 | 42.6 | 46.7 |
| #C@10X (million) | 1.43 | 1.51 | NA | 1.14 | 1.57 | 1.41 | 1.36 | 1.42 |
| Correlation with 450K chip (R^2^) | 0.95 | 0.94 | NA | 0.94 | 0.94 | 0.71 | 0.91 | 0.89 |

**Additional Figure 1: Correlation plot between 450K array and RRBS from comparison tool for MCF7**

| 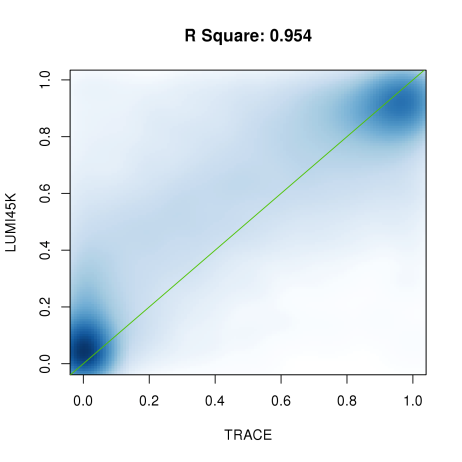 | 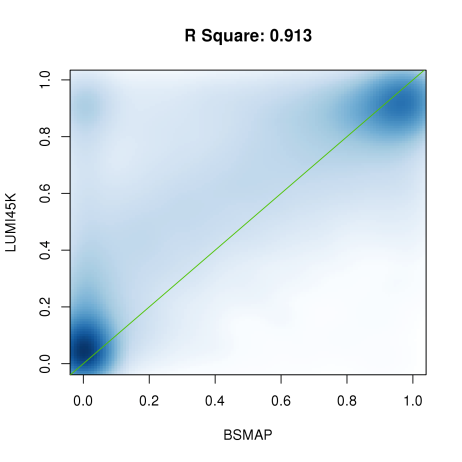 | 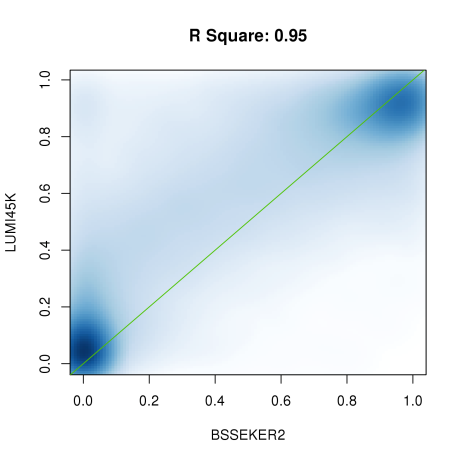 |
| --- | --- | --- |
| 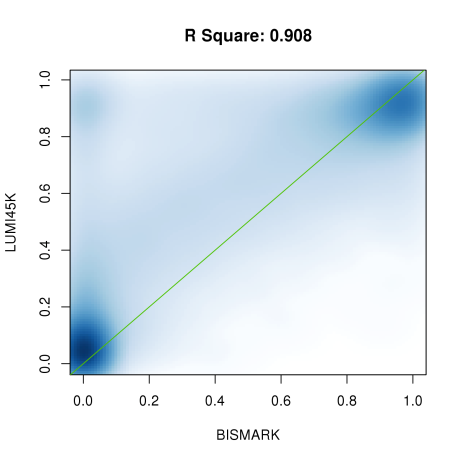 | 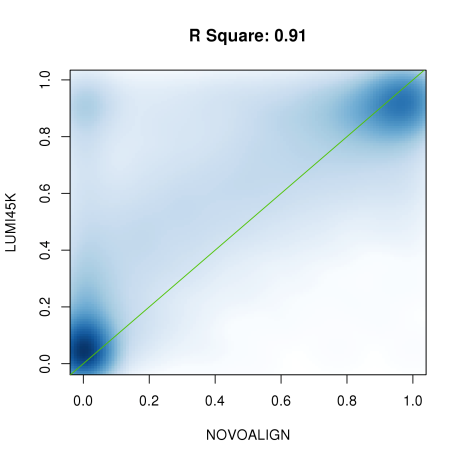 | 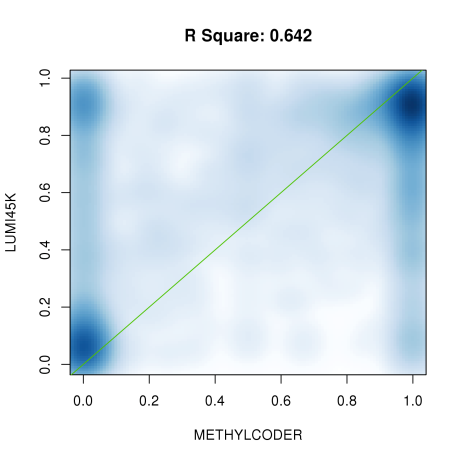 |
| 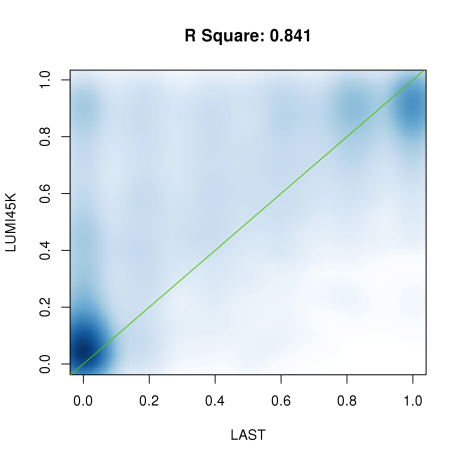 |  |  |
